# Impaired dynamic cerebral autoregulation is associated with the severity of neuroimaging features of cerebral small vessel disease

**DOI:** 10.1111/cns.13778

**Published:** 2021-12-11

**Authors:** Zhaojun Liu, Hongyin Ma, Zhen‐Ni Guo, Le Wang, Yang Qu, Lei Fan, Xingliang Liu, Jie Liu, Yuanyuan Zhu, Yi Yang

**Affiliations:** ^1^ Stroke Center & Clinical Trial and Research Center for Stroke, Department of Neurology The First Hospital of Jilin University Changchun China; ^2^ China National Comprehensive Stroke Center Changchun China; ^3^ Jilin Provincial Key Laboratory of Cerebrovascular Disease Changchun China; ^4^ Department of Neurology The First Hospital of Hebei North University Zhangjiakou China; ^5^ Department of Neurology The People’s Hospital of Lixin County Haozhou China

**Keywords:** cerebral small vessel disease, dynamic cerebral autoregulation, total CSVD burden score, transfer function analysis

## Abstract

**Aims:**

Cerebral small vessel disease (CSVD) is characterized by functional and structural changes in small vessels. We aimed to elucidate the relationship between dynamic cerebral autoregulation (dCA) and neuroimaging characteristics of CSVD.

**Methods:**

A case‐control study was performed. Cerebral blood flow velocity (CBFV) of bilateral middle cerebral arteries and spontaneous arterial blood pressure were simultaneously recorded. Transfer function analysis was used to calculate dCA parameters (phase, gain, and the rate of recovery of CBFV [RoRc]). Neuroimaging characteristics of CSVD patients were evaluated, including lacunes, white matter hyperintensities (WMH), cerebral microbleeds (CMBs), perivascular spaces (PVS), and the total CSVD burden.

**Results:**

Overall, 113 patients and 83 controls were enrolled. Compared with the control group, the phase at low frequency and the RoRc in CSVD patients were lower, and the gain at very low and low frequencies were higher, indicating bilaterally impaired dCA. Total CSVD burden, WMH (total, periventricular and deep), severe PVS, and lobar CMBs were independently correlated with the phase at low frequency.

**Conclusions:**

Our findings suggested that dCA was compromised in CSVD patients, and some specific neuroimaging characteristics (the total CSVD burden, WMH, severe PVS and lobar CMBs) might indicate more severe dCA impairment in CSVD patients.

## Graphical abstract

This is the first study to investigate the relationship between dCA function and comprehensive neuroimaging characteristics of CSVD. The findings linked dCA impairment with cerebral small vessel injuries and proposed that dCA monitoring may help evaluate the small vessel function and provide novel therapeutic targets.

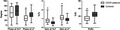

## 1 INTRODUCTION

Cerebral small vessel disease (CSVD) is a collection of clinical, cognitive, neuroimaging, and neuropathological findings associated with changes to the cerebral small vessels and the resulting brain damage in the white and gray matter.^1^ Neuroimaging features of CSVD include recent small subcortical infarcts, lacunes, white matter hyperintensities (WMH), perivascular spaces (PVS), cerebral microbleeds (CMBs) and brain atrophy,^2^ and identification of these features improves the efficacy of neuroimaging examinations in the clinical diagnosis and pathological study of CSVD. CSVD is very common among older adults and contributes substantially to stroke, cognitive decline, depression, and physical disabilities.^3^ However, the complete pathogenesis of CSVD is still enigmatic, which involves endothelial damage, increased BBB permeability, luminal narrowing, and vessel wall thickening and stiffness, indicating that both structural and functional changes of cerebral small vessels are implicated in the pathology of CSVD.^1^, ^3^, ^4^, ^5^ Clinical neuroimaging findings may pragmatically recognize the structural features of the brain lesions and quantify the severity of CSVD,^1^ but the underlying functional alterations of cerebral vasculature cannot be easily determined.

Dynamic cerebral autoregulation (dynamic CA, dCA) refers to the cerebral vasculature's transient response to rapid changes of blood pressure to maintain the stabilization of cerebral blood flow (CBF), and it serves as an indicator of cerebral vasculature function.^6^, ^7^ Using transcranial Doppler (TCD), dCA can be readily measured and analyzed non‐invasively without rigid patient cooperation. Since the reduced effectiveness of dCA capability may jeopardize the brain in the face of the turbulence of blood pressure,^8^ dCA is of significance in a wide range of cerebral pathological settings, including CSVD.^7^, ^9^, ^10^, ^11^, ^12^, ^13^

Until now, several studies have revealed dysfunctions of cerebral hemodynamics or dysautoregulation in CSVD patients.^7^, ^9^, ^10^, ^11^, ^12^, ^13^ For instance, Brickman et al.^11^ found that WMH was correlated with impaired cerebrovascular hemodynamics. Our previous work found that dCA impairment was sustained in patients with lacunar stroke.^7^ However, it is noteworthy that most studies solely focused on one specific subset of patients independently according to neuroimaging feature. Since these features are sometimes independent, but more likely overlapping, concomitant, or inter‐related,^14^ merely focusing on one subset of features seems insufficient to characterize the general CSVD population. Considering this large group of patients with highly variable features, exploring dCA characteristics comprehensively in this population is imperative.

Furthermore, little is known about the correlation of dCA characteristics with diverse neuroimaging features. Which types or locations of lesions as well as the severity of burdens are correlated with dCA impairment? Addressing these questions may link pathology‐imaging correlation and further reveal the pathogenic characteristics of CSVD from a new perspective. Moreover, the clinical significance of exploring dCA in CSVD patients is to remind healthcare practitioners to be aware of the functional changes behind small vessel injuries, and further to optimize the therapeutic strategies based on individual dCA function in clinical practice, such as using CA‐oriented antihypertension therapy in CSVD patients.

In this current study, we aimed to characterize dCA function in CSVD patients. Autoregulatory parameters were obtained using transfer function analysis (TFA) to assess dCA function, including phase, gain, and the rate of recovery of CBFV (RoRc). Further, correlations between dCA and neuroimaging characteristics of CSVD, including lacunes, PVS, CMBs, WMH, and the total CSVD burden score were explored.

## 2 METHODS

### 2.1 Participants and clinical assessment

We performed a case‐control study of consecutive admissions to the Department of Neurology at the First Hospital of Jilin University, the First Hospital of Hebei North University, and the People's Hospital of Lixin County, from December 2016 to October 2020. Patients were eligible for inclusion in this study if they met the following criteria: (1) they were diagnosed with CSVD by at least two neurologists and presented with at least one magnetic resonance imaging (MRI) feature of CSVD that met the STandards for ReportIng Vascular changes on nEuroimaging (STRIVE);^2^ (2) the bilateral temporal bone windows could be well penetrated for TCD insonation; (3) they had a complete set of MRI images including T1‐weighted, T2‐weighted, and diffusion‐weighted imaging (DWI), susceptibility‐weighted imaging (SWI), and fluid‐attenuated inversion recovery (FLAIR) imaging; and (4) they were conscious and able to cooperate sufficiently to complete the clinical examinations and dCA evaluation. Patients with (1) a history of other cerebrovascular diseases (such as stroke, transient ischemic attack, etc.) within 3 months, (2) moderate‐to‐severe carotid or intracranial artery stenosis (≥50%) or occlusion, or (3) myocardial infarction, atrial fibrillation, heart failure, autonomic nervous disorder, or other diseases that could affect cerebral hemodynamics were excluded from this study. In addition, individuals recruited from the public who presented no MRI features of CSVD as well as met the above exclusion criteria were included as the control group. To ensure that both the age‐ and sex‐distribution of the control group was consistent with that of the CSVD group, controls were recruited with ±5‐year deviation in age and matched sex distribution.

Demographic and clinical data were collected, including sex, age, current smoking or excessive drinking status, history of hypertension, hyperlipidemia and diabetes, and baseline medication regimens (antihypertensive treatment, statin, antiplatelet treatment, and antidiabetic treatment). In addition, the following clinical laboratory examination results were collected: fasting blood glucose, total cholesterol, triglyceride, low‐density lipoprotein cholesterol, and urid acid levels.

This study followed the guidelines of the Declaration of Helsinki, and was approved by the Ethics Committee of the First Hospital of Jilin University, the First Hospital of Hebei North University, and the People's Hospital of Lixin County. Written informed consent was obtained from all participants before the investigation.

### 2.2 MRI assessment

**FIGURE 1:**
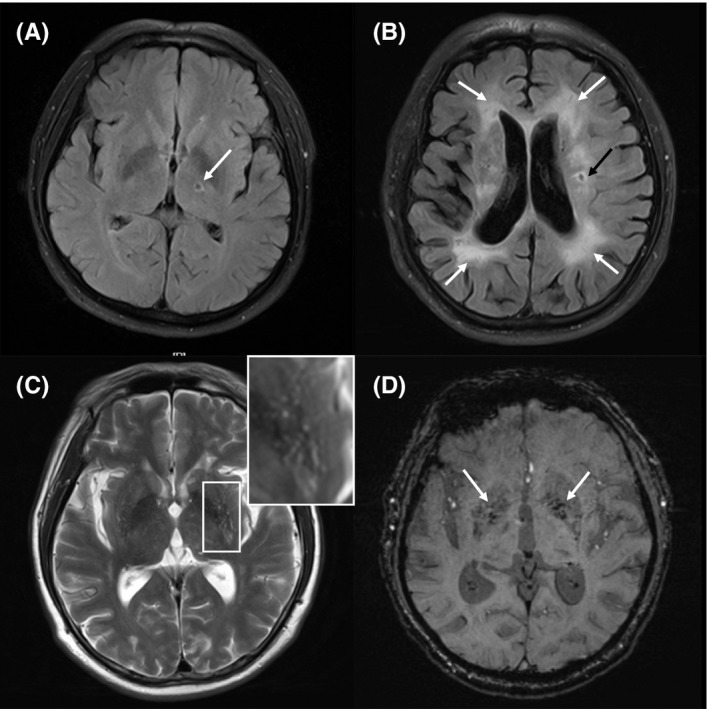
Representative MRI features of CSVD. (A) Lacune (arrow) on fluid‐attenuated inversion recovery (FLAIR) imaging. (B) White matter hyperintensities (white arrows) accompanied by lacune (black arrow) on FLAIR imaging. (C) Perivascular spaces on T2‐weighted imaging. (D) Cerebral microbleeds (arrows) on susceptibility‐weighted imaging (SWI)

All participants underwent brain MRI scanning using 3.0‐T MRI scanners (Philips Ingenia). Representative images of characteristic CSVD lesions are shown in Figure 1. MRI images were reviewed by at least two trained neurologists who were blinded to the study information. Each component of CSVD neuroimaging features was recorded and rated according to the STRIVE standard.^2^ If different scores were given, the relevant MRI images would be discussed among at least 3 authors blindly, and the final score was recorded if the consensus was once achieved. For WMH, the Fazekas score (from 0 to 3 points) was given for periventricular WMH (PWMH) and deep WMH (DWMH) separately, based on FLAIR images, and was then summed into a total WMH burden score.^15^ PVS in the basal ganglia are known to be closely connected with other CSVD.^16^ Therefore, the number of PVS in the basal ganglia was rated on T2‐weighted images as follows^17^: 0 = absent, 1 = 1–10 PVS, 2 = 11–20 PVS, 3 = 21–40 PVS, and 4 = more than 40 PVS. The severity of PVS was classified into four categories (0, absent; 1, mild; 2, moderate; and 3–4, severe). The topographic distribution of CMBs in the brain was classified as lobar and deep on SWI images.^18^, ^19^ The number of lobar and deep CMBs was recorded, respectively, as follows^20^: 0 = absent, 1 = single CMB, 2 = 2–4 CMBs, and 3 = more than 4 CMBs. The severity of CMBs was then divided into three categories (0, absent; 1–2, mild to moderate; 3, severe). The total CSVD burden score is the summed score for each of the four imaging features: lacunes, CMBs, PVS (grade 2–4 in the basal ganglia), and WMH (defined as Fazekas 3 for PWMH and/or Fazekas 2–3 for DWMH). The score ranged from 0 to 4 points.^21^

### 2.3 Measurement of dCA

DCA measurement was performed after confirming the diagnosis of CSVD. Before the measurement, participants were asked to avoid alcohol, caffeinated drinks, and exercise for at least 12 h and to avoid chocolate and heavy meals for at least 4 h. The measurement was performed in a dedicated laboratory with temperature controlled at 22–24°C where both visual and auditory stimuli were minimized. The measurement was performed by a physician specialized in neurovascular ultrasound who was blinded to the clinical information of all the participants. To ensure the accuracy of the dCA data, the participants were required to adopt a relaxed supine position for 15 min before the measurement. The baseline blood pressure was measured at the left brachial artery using an automatic blood pressure monitor (Omron 711). A 2‐MHz TCD (MultiDop X2, DWL; or EMS‐9A, Delica) was placed at bilateral anterior temporal windows to record the cerebral blood flow velocity (CBFV) of bilateral middle cerebral arteries (MCA), combined with a servo‐controlled plethysmograph (Finometer Pro) to simultaneously record continuous arterial blood pressure (ABP). The end‐tidal CO_2_ level was monitored and recorded using a capnograph with a face mask attached to the nasal cannula. To obtain uninterrupted high‐quality data, all participants were asked to stay awake and to avoid talking and body movements for at least 5 min to maintain physiological conditions during the measurement.

### 2.4 Analysis of dCA data

The dCA data were analyzed by dedicated data processing personnel using MATLAB (MathWorks). A cross‐correlation function was used to achieve beat‐to‐beat alignment, which is critical for time‐lag elimination. A third‐order Butterworth low‐pass filter (cutoff at 0.5 Hz) was used as an anti‐aliasing filter before down‐sampling the data to 1 Hz. The autoregulatory parameters of each participant (phase, gain, and coherence), calculated by TFA, were obtained separately within two main frequency ranges, as previously reported: very low frequency (VLF, 0.02–0.07 Hz) and low frequency (LF, 0.07–0.20 Hz).^22^, ^23^ The phase provides a measure of the temporal difference between CBFV oscillations in relation to ABP (a lower phase indicates impaired dCA). Meanwhile, the gain quantifies the damping effect of CA on the magnitude of oscillations in the blood pressure (a higher gain indicates impaired dCA). The coherence reflects the degree of the linear relationship between CBFV oscillations and ABP. In the time domain, the step response of CBFV reflects the recovery of CBF after a stepwise change in ABP, and the rate of recovery of CBFV (RoRc) (within the first 3 s of the response), was defined as ΔCBF/Δ*t* × 100% (a lower RoRc indicates impaired dCA).^24^

### 2.5 Statistical analysis

All collected data were analyzed using SPSS 26.0 (SPSS, IBM). For continuous variables, the Shapiro‐Wilk test was performed to determine the normality of the data distribution. Continuous variables with normal distribution are presented as mean ± standard deviation, while those with non‐normal distribution are presented as median (interquartile range). Categorical variables are reported as the rate (percentage). To compare differences in clinical and physiological characteristics between CSVD patients and healthy controls, the Student's *t*‐test or the Mann‐Whitney *U* test was used for continuous variables, and the χ^2^ test was used for categorical variables.

The Wilcoxon signed‐rank test was used to compare the bilateral differences in dCA parameters of CSVD patients, including phase, gain (at corresponding frequency bands), and RoRc. In addition, the mean phase, gain, and RoRc of bilateral hemispheres were calculated for further analyses. The Mann‐Whitney *U* test was used to compare the differences in the above‐mentioned dCA parameters between CSVD patients and healthy controls. The univariable and multivariable linear regression analyses were used to explore the correlations between dCA parameters and neuroimaging characteristics of CSVD (lacunes, WMH, CMBs, PVS, and the total CSVD burden score). Statistical significance was set at *p*‐values <0.05.

## 3 RESULTS

**FIGURE 2:**
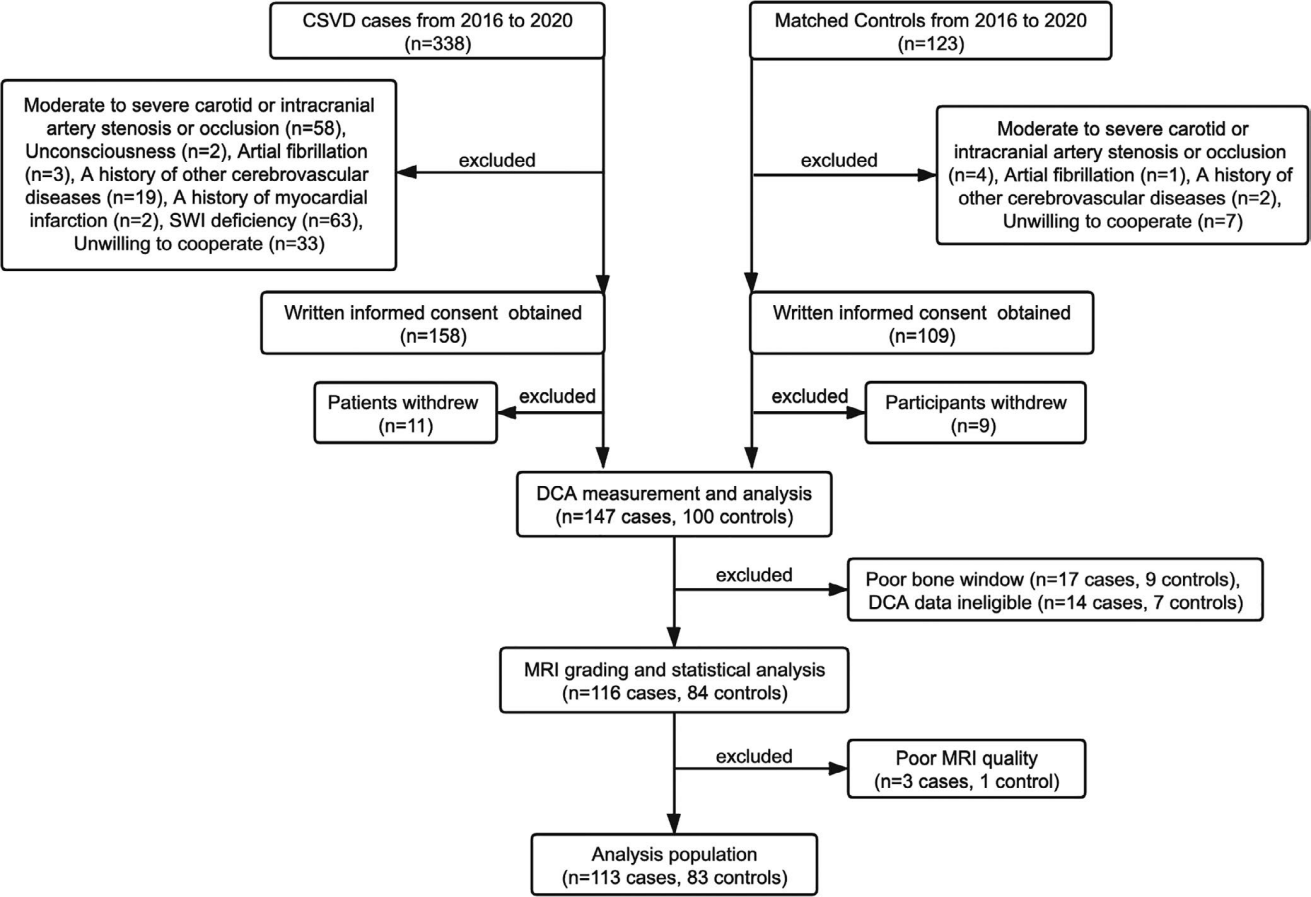
Flow chart of the study

**FIGURE 3:**
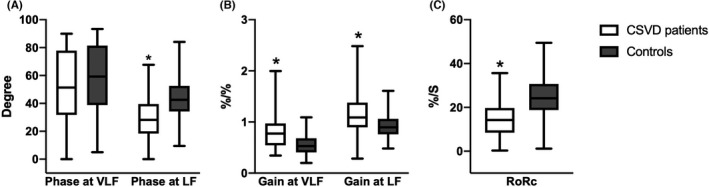
DCA parameters in patients with CSVD compared with controls. Box‐and‐whisker plots of (A) Phase in patients with CSVD and controls, at very low frequency (VLF, 0.02–0.07 Hz) and low frequency (LF, 0.07–0.20 Hz) bands, respectively. (B) Gain in patients with CSVD and controls, at VLF and LF bands, respectively. (C) The rate of recovery of CBFV (RoRc) in patients with CSVD and controls. ∗ denotes *p* < 0.05 for comparison with controls (at corresponding frequency bands)

Figure 2 shows the flow chart of this study. A total of 113 CSVD patients and 83 age‐ and sex‐matched controls were included in this study. The clinical and neuroimaging baseline characteristics of the patients with CSVD are presented in Table 1. There was no significant difference in the distribution of sex, age, current smoking or excessive drinking status, or the prevalence of diabetes between patients with CSVD and healthy controls. However, the prevalence of hypertension and hyperlipidemia in the patients with CSVD was higher than that in the control group. During dCA measurements, the mean arterial blood pressure (MAP) of CSVD patients was higher than that of healthy controls, and the mean CBFV of bilateral MCA in CSVD patients was lower than that in healthy controls. Other physiological parameters were not significantly different between the CSVD and the control group (Table 2). Next, the dCA parameters were compared between the CSVD and the control group (Figure 3).

**TABLE 1:** The clinical and neuroimaging characteristics of patients with CSVD

	CSVD patients (*n* = 113)	Controls (*n* = 83)	*p*
Age (years)	56.01 ± 10.62	53.65 ± 9.27	0.202
Sex (male/female)	92/21	69/14	0.757
Current smoking	74 (65.5)	45 (42.2)	0.110
Excessive drinking	47 (41.6)	26 (31.3)	0.142
Hypertension	68 (60.2)	33 (39.8)	0.005^*^
Diabetes	27 (23.9)	11 (13.3)	0.063
Hyperlipidemia	67 (59.3)	34 (41.0)	0.011^*^
Antihypertensive treatment	25 (22.1)		
Statin	31 (27.4)		
Antiplatelet treatment	42 (37.2)		
Antidiabetic treatment	14 (12.4)		
FBG (mmol/L)	5.22 (4.81–6.26)		
Triglyceride (mmol/L)	1.57 (1.12–2.91)		
Total cholesterol (mmol/L)	4.87 ± 1.00		
LDL‐C (mmol/L)	2.89 ± 0.66		
Urid acid (mmol/L)	335.0 (283.0–389.0)		
Lacunes	78 (69.0)		
PWMH	64 (56.6)		
DWMH	44 (38.9)		
PVS	81 (71.7)		
Lobar CMBs	17 (15.0)		
Deep CMBs	27 (23.9)		
Total CSVD burden score			
0	23 (20.4)		
1	37 (32.7)		
2	31 (27.4)		
3	10 (8.8)		
4	12 (10.6)		

Abbreviations: CMBs, cerebral microbleeds; CSVD, cerebral small vessel disease; DWMH, deep white matter hyperintensities; FBG, fasting blood glucose; LDL‐C, low‐density lipoprotein cholesterol; PVS, perivascular spaces; PWMH, periventricular white matter hyperintensities.

∗ *p* < 0.05 for comparison with controls.

**TABLE 2:** Physiological data of patients with CSVD and healthy controls during dCA measurements

	CSVD patients (*n* = 113)	Healthy controls (*n* = 83)
MAP (mmHg)	102.6 ± 14.2*	85.8 ± 10.6
Heart rate (beats/min)	67 (63–74)	67 (64–78)
Mean CBFV (cm/s)	63.2 (54.4–71.9)*	68.5 (62.8–82.8)
EtCO_2_ (mmHg)	37.9 ± 1.4	38.0 ± 1.4

Abbreviations: CBFV, cerebral blood flow velocity; EtCO_2_, end‐tidal CO_2_; MAP, mean arterial blood pressure.

* *p* < 0.05 for comparison with controls.

### 3.1 DCA parameters in CSVD patients vs. controls

We first investigated whether there was any difference in the bilateral dCA parameters of CSVD patients, and found that there was no significant difference in the bilateral dCA parameters, including phase, gain (at corresponding frequency bands), and RoRc (Table S1). Thereafter, the mean phase, gain, and RoRc of bilateral hemispheres were calculated for further analyses.

#### 3.1.1 Phase

The phase provides a measure of the temporal difference between CBFV oscillations in relation to ABP (a lower phase indicates impaired dCA). Compared with the control group, the phase at LF in the CSVD group was significantly lower (CSVD vs. control, 27.05 [18.39–39.92] vs. 42.56 [34.24–52.60], *p* < 0.001). Furthermore, no significant difference for the phase at VLF was noted; however, there was a tendency toward lower phase at VLF in the CSVD group (CSVD vs. control, 53.33 [34.83–79.18] vs. 59.48 [39.05–81.87], *p* = 0.103).

#### 3.1.2 Gain

The gain quantifies the damping effect of CA on the magnitude of oscillations in the blood pressure (a higher gain indicates impaired dCA). The gain at both VLF and LF in the CSVD group were significantly higher than those in the control group (gain at VLF: CSVD vs. control, 0.79 [0.54–1.02] vs. 0.53 [0.41–0.69], *p* < 0.001; gain at LF: CSVD vs. control, 1.12 [0.93–1.39] vs. 0.90 [0.79–1.07], *p* < 0.001, respectively).

#### 3.1.3 RoRc

The RoRc was obtained to quantify the efficiency of the step response (a lower RoRc indicates impaired dCA). The RoRc was significantly lower in the CSVD group than in the control group (CSVD vs. control, 14.87 [9.88–20.10] vs. 24.15 [18.77–30.70], *p* < 0.001).

### 3.2 Relationship between dCA and neuroimaging characteristics of CSVD

The phase at LF was statistically associated with clinical factors and neuroimaging characteristics of CSVD in linear regression analyses, while other dCA parameters were not found to be relevant. In univariable linear regression analyses, heart rate was found to have a significant linear correlation with the phase at LF among all the clinical characteristics (*β* = 0.403, *p* = 0.013) (Table S2); meanwhile, total CSVD burden, WMH (total, DWMH and PWMH), severe PVS, and lobar CMBs were found to have significant linear correlations with the phase at LF, respectively (Figure S1). Variables according to clinical consideration and those with *p*‐values ≤0.1 in the univariable analyses were then introduced into the following multivariable linear regression analyses. In multivariable linear regression analyses, the total CSVD burden score (*β* = −3.363, *p* = 0.009), total WMH (*β* = −2.437, *p* = 0.002), DWMH (*β* = −4.427, *p* = 0.003), PWMH (*β* = −4.256, *p* = 0.006), lobar CMBs (*β* = −7.358, *p* = 0.017), and severe PVS (*β* = −11.015, *p* = 0.040) were independently correlated with the phase at LF (Table 3).

**TABLE 3:** Multivariable linear regression analyses between neuroimaging characteristics and the phase at LF

	The phase at LF			The phase at LF	
	*β*	*p*		*β*	*p*
Sex	−3.125	0.456	Sex	−3.787	0.360
Age	−0.084	0.580	Age	−0.081	0.585
HR (beats/min)	0.408	0.011*	HR (beats/min)	0.440	0.006*
Total CSVD	−3.363	0.009*	Total WMH	−2.437	0.002*
	*β*	*p*		*β*	*p*
Sex	−4.708	0.258	Sex	−2.858	0.494
Age	−0.120	0.412	Age	−0.069	0.647
HR (beats/min)	0.429	0.007*	HR (beats/min)	0.436	0.007*
DWMH	−4.427	0.003*	PWMH	−4.256	0.006*
	*β*	*p*		*β*	*p*
Sex	−3.268	0.439	Sex	−3.118	0.464
Age	−0.162	0.270	Age	−0.173	0.243
HR (beats/min)	0.458	0.006*	HR (beats/min)	0.340	0.036*
Lobar CMBs	−7.358	0.017*	Severe PVS	−11.015	0.040*

Abbreviations: CMBs, cerebral microbleeds; CSVD, cerebral small vessel disease; DWMH, deep white matter hyperintensities; HR, heart rate; PVS, perivascular spaces; PWMH, periventricular white matter hyperintensities; WMH, white matter hyperintensities.

* Denotes *p* < 0.05 in multivariable linear regression analyses.

## 4 DISCUSSION

The present study has shown that dCA was bilaterally impaired in patients with CSVD, and there were negative associations between dCA function and the total CSVD burden score, the severity of WMH, lobar CMBs, and PVS. The present findings link dCA impairment and cerebral small vessel injuries, and suggest that some specific neuroimaging characteristics might indicate more severe dCA impairment in CSVD patients.

The CSVD is prevalent in the elderly. Almost all people older than 90 years old exhibit clinical or radiologic manifestations of CSVD.^25^, ^26^ Until recently, the specific pathological mechanisms underlying CSVD still remained largely unknown, in part because of the lack of effective methods that can technically visualize or image small vessels in vivo.^4^, ^25^ Although the advances in neuroimaging have provided novel information in CSVD including detailed evaluation of the vessel wall, atherosclerotic plaques within intracranial arteries,^27^, ^28^ white matter integrity, WMH shape, and the fraction of free water in the drainage area,^29^, ^30^, ^31^ there have been few researches investigating the dynamic vasculature's function of small vessels, such as dCA.

Several studies have examined the relationship between CSVD and dCA. In our previous studies, we found that the impairment of dCA in patients with lacunar infarction was diffuse and sustained, creating a foundation for the present study.^7^, ^9^ In addition, the characteristics of CA in patients with WMH have also been previously identified; however, these findings were inconsistent. In 1994, Matsushita et al.^10^ studied the relationship between CA and WMH in patients with chronic hypertension, and found that patients with more severe periventricular lesions were more likely to have CA impairment. Moreover, Brickman et al.^11^ also reported a positive relationship between the volume of WMH and dCA impairment, in which Aβ deposition may play a role. In contrast, while measuring posterior cerebral circulation, overreactive (rather than impaired) CA was associated with WMH severity.^32^ However, other studies failed to report correlations between CA and WMH.^33^, ^34^ Such inconsistences might be explained by the differences in study designs, and methods or measurements used. Regarding other CSVD neuroimaging features, both CMBs and brain atrophy have been shown to be associated with impaired CA.^12^, ^13^

Compared with most of the previous studies, our study contains a larger sample size and more comprehensive neuroimaging features. Our study found that the phase at LF and RoRc were lower and the gain at both VLF and LF were higher in the CSVD group, demonstrating that dCA was impaired bilaterally in CSVD patients. Although no statistically significant difference was found in the phase at VLF, there was a decreasing trend in the CSVD group, further supporting the observation that dCA was impaired bilaterally in the CSVD group. Our study also revealed that the degree of the impairment was positively associated with the severity of CSVD neuroimaging features, including the total CSVD burden, WMH, PVS, and lobar CMBs. The more severe the neuroimaging features, the more impaired the dCA.

There are several clinical and scientific merits of our study. First, our study observed bilateral CA impairment in patients with CSVD, which is in line with the recently conclusion that CSVD is considered as a ‘whole‐brain’ disease rather than a localized or isolated condition. Second, after confirming the linear relationship between dCA and CSVD neuroimaging features, we propose dCA monitoring as a feasible method for investigating the dynamic vasculature's function of small vessels, which would supplement the limited neuroimaging techniques used in clinical practice. Third, little consensus has been achieved on the question of where exactly CA takes place, and both small pial arterioles and large arteries were recently thought to serve in this process.^35^, ^36^ However, human studies that explore this kind of problem are scarce. The findings of our present study support the significant role of small vessels in CA, as we excluded patients with moderate‐to‐severe large artery stenosis or occlusion, thus eliminating the impact of large artery stenosis or occlusion on CA impairment.

Regarding the prevention and treatment of CSVD, despite its high prevalence, there have been few reliable therapeutic strategies.^26^, ^37^ As hypertension is the strongest known vascular risk factor for CSVD, the dCA capability after antihypertensive therapy is indeed a topic worth discussing.^37^ Theoretically, an effective long‐term blood pressure management may potentially protect dCA in patients with CSVD due to the improvement of constructional deterioration and cerebral vasculature associated with dCA. However, until now, studies about the impact of antihypertensive therapy on CA are mainly on the stage of preliminary animal experiments, indicating that some specific drugs may protect or improve CA.^38^, ^39^, ^40^, ^41^, ^42^ Clinical trials or studies of blood pressure management aiming at improving CA as a treatment target are warranted in CSVD patients. As a feasible method for evaluating cerebral hemodynamics, real‐time bedside CA monitoring has been applied to direct individual cerebral perfusion pressure (CPP) and blood pressure management. For example, Diedler et al.^43^ found that mortality was the lowest in the group of cerebral hemorrhage patients with an actual CPP close to optimal CPP. Rasulo et al.^44^ also proved that the evaluation and direction of CA may provide important information regarding long‐term outcome. Thus, CA‐oriented blood pressure management may become one of the feasible antihypertensive strategies in CSVD patients, which needs to be verified by more sufficient preclinical and clinical evidence in the near future.

The mechanism of CA impairment in patients with CSVD remains unclear. We propose two possible explanations related to endothelial dysfunction that may help understand the relationship between CSVD and CA. First, the regulation of myocyte contractile tone is mediated by vasomotor factors expressed by endothelial cells, including NO and ET‐1^45^; therefore, the abnormal expression of such factors related to CSVD might account for the presence of CA impairment.^45^, ^46^, ^47^, ^48^ Second, the loss of endothelial integrity may lead to the leakage of toxins and plasma components into the sub‐endothelial layers, causing vessel wall swelling, narrowing of lumen, and smooth muscle damage, which may lead to CA impairment.^1^, ^49^

Moreover, the two main pathological features of CSVD may also help explain the presence of CA impairment: arteriolosclerosis and cerebral amyloid angiopathy (CAA). Arteriolosclerosis is characterized by the loss of smooth muscle cells, deposits of fibro‐hyaline material, narrowing of lumen, and thickening of the vessel wall,^4^ resulting in rigidity and stiffness of the vessels.^50^ It is suggested that such pathological changes may lead to CA impairment.^1^, ^4^, ^5^ CAA is characterized by the progressive accumulation of amyloid protein in the walls of small‐to‐medium‐sized arteries and arterioles.^4^ The deposition of amyloid proteins has been reported as closely associated with CA impairment.^11^, ^13^ Notably, in the present study, we only observed a negative linear relationship between the severity of lobar CMBs and the phase at LF in multivariable analyses; no such association was observed in the deep CMBs group. This finding provides further evidence that CAA may be associated with CA impairment in CSVD patients, as lobar CMBs were thought to result from CAA.^2^, ^4^, ^51^

The present study has some limitations, which should be considered when interpreting its findings. First, confined by the TCD methodology, elderly patients are more likely to have poor temporal bone windows precluding successful penetration, which may partially explain the reason why the mean age of CSVD patients in our study is lower than that reported in previous studies.^52^, ^53^ Second, the sample size was still relatively small; further studies with larger sample size are required to validate the present findings.

## 5 CONCLUSIONS

Our study indicated that dCA was impaired bilaterally in patients with CSVD, and the extent of the impairment was positively associated with the severity of CSVD neuroimaging features. This study confirmed the role of cerebral small vessels in dCA and proposed that dCA monitoring may help evaluate cerebral small vessel function and provide therapeutic targets.

## CONFLICT OF INTEREST

The authors reported no conflict of interest.

## AUTHOR CONTRIBUTIONS

HM and ZL designed the study. ZL, LW, LF, XL, JL, and YZ performed the data collection. HM, ZL, and YQ performed the data analyses. ZL wrote the manuscript. Z‐N G, HM, and YY critically revised the manuscript.

## 6 DATA AVAILABILITY STATEMENT

The data that support the findings of this study are available from the corresponding author upon reasonable request.

## Supporting information

Fig S1

Supplementary Material
