# Supplementary material for: Impaired dynamic cerebral autoregulation is associated with the severity of neuroimaging features of cerebral small vessel disease
Source: CNS Neurosci Ther. 2021 Dec 11;28(2):298–306. doi: 10.1111/cns.13778 (PMC8739047; doi:10.1111/cns.13778)
Supplement: Supplementary file 2 — Supplementary Material [file CNS-28-298-s001.docx]

**Supplementary Table 1. Bilateral dCA parameters in patients with CSVD**

|  | Left hemisphere | Right hemisphere | *P* |
| --- | --- | --- | --- |
| VLF |  |  |  |
| Phase (^o^) | 52.36 (34.33-80.25) | 50.45 (34.31-82.75) | 0.099 |
| Gain (%/%) | 0.77 (0.54-1.03) | 0.77 (0.52-0.96) | 0.475 |
| LF |  |  |  |
| Phase (^o^) | 28.75 (20.71-42.02) | 27.71 (16.86-39.51) | 0.081 |
| Gain (%/%) | 1.13 (0.93-1.37) | 1.15 (0.91-1.41) | 0.601 |
| RoRc (%/s) | 15.09 (8.51-20.91) | 14.64 (9.22-20.23) | 0.089 |

VLF, very low frequency; LF, low frequency; RoRc, the rate of recovery of CBFV

**Supplementary Table 2. Univariable linear regression analyses between clinical characteristics and the phase at LF**

|  | The phase at LF | |
| --- | --- | --- |
|  | *β* | *P* |
| Sex | -3.857 | 0.364 |
| Age (years) | -0.265 | 0.064^a^ |
| Current smoking | -3.799 | 0.251 |
| Excessive drinking | 4.187 | 0.190 |
| Hypertension | -2.735 | 0.393 |
| Diabetes | -3.947 | 0.302 |
| Hyperlipidemia | -1.214 | 0.706 |
| Heart rate（beats/min） | 0.403 | 0.013^ab^ |
| MAP（mmHg） | 0.090 | 0.251 |
| FBG（mmol/L） | -0.500 | 0.471 |
| LDL-C（mmol/L） | 0.450 | 0.859 |
| Triglyceride（mmol/L） | -1.141 | 0.331 |
| Total cholesterol（mmol/L） | -0.501 | 0.759 |
| Urid acid（mmol/L） | 0.003 | 0.857 |

MAP, mean arterial blood pressure; FBG, fasting blood glucose; LDL-C, low density lipoprotein cholesterol; ^a^ denotes *P* < 0.1 in univariable linear analyses; ^b^ denotes *P* < 0.05 in univariable linear analyses.

**Supplementary Figure Legend:**

**Supplementary Figure 1: Correlations between the phase at LF and the severity of MRI features in patients with CSVD.**

Box-and-whisker plots of the phase at LF in patients with various MRI features of CSVD (A-H). The phase at LF had positive associations with the total CSVD burden (*P* for trend=0.004), total WMH (*P* for trend=0.003), DWMH (*P* for trend=0.005), PWMH (*P* for trend=0.006), severe PVS (*P* for trend=0.018), lobar CMBs (*P* for trend=0.043) in univariable linear regression analyses.
